# TRIM22-mediated ubiquitin-dependent degradation of FLT3-ITD overcomes TKI resistance in acute myeloid leukemia

**DOI:** 10.1093/lifemedi/lnag017

**Published:** 2026-05-14

**Authors:** Meng Liu, Xiaoqian Wang, Xiaqin He, Xiaoguang Xu, Jing Chen, Juan Liu, Shan Jiang, Sijia Li, Si Wang, Yuanyuan Liu, Jing Feng, Xin Xu, Miaoyin Luo, Zhiyi Li, Yining Zhang, Yingli Wu, Jian Hu, Xiaoqin Wang

**Affiliations:** Department of Clinical Laboratory, The First Affiliated Hospital of Xi’an Jiaotong University, Xi’an 710061, China; Department of Clinical Laboratory, The First Affiliated Hospital of Xi’an Jiaotong University, Xi’an 710061, China; Department of Clinical Laboratory, The First Affiliated Hospital of Xi’an Jiaotong University, Xi’an 710061, China; Department of Hematology, The First Affiliated Hospital of Xi’an Jiaotong University, Xi’an 710061, China; Department of Hematology, The First Affiliated Hospital of Xi’an Jiaotong University, Xi’an 710061, China; Department of Hematology, The First Affiliated Hospital of Xi’an Jiaotong University, Xi’an 710061, China; Department of Clinical Laboratory, The First Affiliated Hospital of Xi’an Jiaotong University, Xi’an 710061, China; Department of Clinical Laboratory, The First Affiliated Hospital of Xi’an Jiaotong University, Xi’an 710061, China; Department of Clinical Laboratory, The First Affiliated Hospital of Xi’an Jiaotong University, Xi’an 710061, China; Department of Clinical Laboratory, The First Affiliated Hospital of Xi’an Jiaotong University, Xi’an 710061, China; Department of Clinical Laboratory, The First Affiliated Hospital of Xi’an Jiaotong University, Xi’an 710061, China; Department of Clinical Laboratory, The First Affiliated Hospital of Xi’an Jiaotong University, Xi’an 710061, China; Department of Clinical Laboratory, The First Affiliated Hospital of Xi’an Jiaotong University, Xi’an 710061, China; Department of Hematology, The First Affiliated Hospital of Xi’an Jiaotong University, Xi’an 710061, China; Department of Blood Transfusion, The First Affiliated Hospital of Xi’an Jiaotong University, Xi’an 710061, China; Hongqiao International Institute of Medicine, Shanghai Tongren Hospital/Faculty of Basic Medicine, Chemical Biology Division of Shanghai Universities E-Institutes, Key Laboratory of Cell Differentiation and Apoptosis of the Chinese Ministry of Education, Shanghai Jiao Tong University School of Medicine, Shanghai 200025, China; Department of Clinical Laboratory, The First Affiliated Hospital of Xi’an Jiaotong University, Xi’an 710061, China; Department of Clinical Laboratory, The First Affiliated Hospital of Xi’an Jiaotong University, Xi’an 710061, China

**Keywords:** TRIM22, FLT3-ITD, ubiquitin, acquired resistance, APG-115

## Abstract

FMS-like tyrosine kinase 3 internal tandem duplication (FLT3-ITD) mutations, found in 25%–30% of acute myeloid leukemia (AML) patients, cause poor prognosis and resistance to FLT3 tyrosine kinase inhibitors (TKIs). We show that APG-115 selectively kills FLT3-ITD cells by activating p53. This critically upregulates TRIM22 (Tripartite motif-containing protein 22), an essential E3 ubiquitin ligase that directly binds FLT3-ITD, promotes its polyubiquitination, and induces its proteasomal degradation. This TRIM22-mediated mechanism offers a novel strategy to overcome intrinsic and acquired TKI resistance. Unlike inhibitors such as AC220, which suppress FLT3 signaling but downregulate p53, APG-115 restores p53 function and induces TRIM22, enabling potent synergy with FLT3 inhibitors. TRIM22 is essential for APG-115’s suppression of leukemia stem cells, inducing cell cycle arrest, myeloid differentiation, and reduced clonogenic potential. The combination of APG-115 and AC220 significantly enhances apoptosis in FLT3-ITD AML models and primary cells, while sparing normal cells. It shows robust efficacy in preclinical xenograft models, reducing tumor burden and extending survival. This work establishes targeting the p53/TRIM22 axis, reliant on TRIM22’s unique activity against FLT3-ITD, as a highly promising therapeutic approach for FLT3-ITD AML, including resistant disease.

## Introduction

Acute myeloid leukemia (AML) harboring FMS-like tyrosine kinase 3 internal tandem duplication (FLT3-ITD) mutations represents a clinically aggressive subtype characterized by high relapse rates and poor survival outcomes despite advances in targeted therapies [1–3]. The constitutive activation of FLT3 tyrosine kinase drives aberrant proliferation and survival of leukemic blasts, while resistance to FLT3 inhibitors (TKI) such as sorafenib and gilteritinib remains a major therapeutic hurdle [4–6]. Emerging evidence suggests that resistance mechanisms involve not only secondary FLT3-TKD mutations (e.g. D835Y) but also adaptive activation of parallel survival pathways, including FOXO-mediated upregulation of HDAC8, resulting in p53 deacetylation and functional inactivation [7, 8], as well as TRIM28/MDM2 complex-mediated promotion of p53 ubiquitination and degradation [9]. However, the bidirectional regulatory relationship between FLT3 signaling and p53, and its role in therapeutic resistance, remains a significant knowledge gap.

The dual targeting of FLT3 and p53 pathways presents a promising therapeutic strategy, as p53 restoration could simultaneously disrupt FLT3-ITD stability and eliminate leukemia stem cells (LSCs), a critical reservoir for relapse [10–12]. While MDM2 inhibitors like APG-115 are known to stabilize p53, their role in modulating FLT3-ITD protein turnover remains unexplored. Our preliminary data reveal that APG-115 induces FLT3-ITD degradation via the E3 ubiquitin ligase TRIM22, a novel p53 effector, suggesting a mechanistic link between p53 activation and FLT3-ITD suppression. This contrasts with conventional TKIs, which primarily inhibit kinase activity without affecting protein abundance [13].

TRIM22, a member of the tripartite motif protein family with multifaceted roles in viral restriction, immune regulation, and carcinogenesis [14–16] and an interferon-stimulated gene (ISG) regulated by p53 [17–19], has been reported to possess E3 ubiquitin ligase activity. As a direct p53 target gene, TRIM22 mediates p53-dependent growth inhibition in leukemia cells and is downregulated in breast cancer due to impaired p53 transactivation [20, 21]. Conversely, TRIM22 exerts oncogenic functions in non-small cell lung cancer (NSCLC) by activating AKT/GSK3β/β-catenin signaling to drive epithelial–mesenchymal transition and metastasis [22]. Despite these advances, TRIM22’s role in hematologic malignancies, particularly its interplay with oncogenic drivers like FLT3-ITD in AML, remains underexplored.

Here, we found that p53 and FLT3-ITD engage in reciprocal regulatory control: Inhibition of FLT3-ITD signaling (e.g. by AC220) induces reduction of p53 protein, suppressing its tumor-suppressor function. Conversely, pharmacologic stabilization of p53 by APG-115 triggers transcriptional upregulation of TRIM22, which directly binds FLT3-ITD as an E3 ubiquitin ligase, catalyzing its polyubiquitination and degradation. Critically, combining APG-115 with FLT3 inhibitor AC220 synergistically enhances apoptosis *in vitro* and significantly reduces tumor burden and extends survival *in vivo*, providing a novel therapeutic strategy to overcome FLT3 inhibitor resistance in AML patients.

## Results

### The MDM2–p53 interaction inhibitor exhibits specific inhibitory effects on FLT3-ITD AML cells

**Figure 1.**
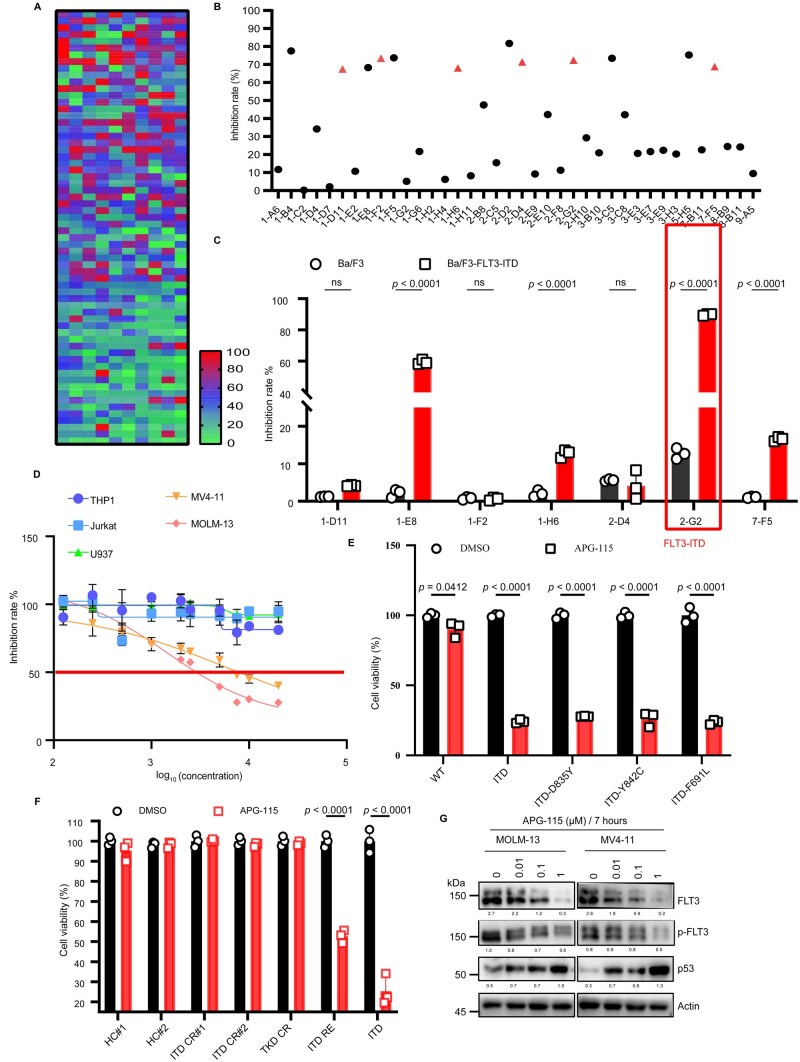
APG-115 exerts potent and selective anti-tumor activity against FLT3-ITD AML cells and induces FLT3-ITD degradation. (A) 0.2 μL of each 10 mmol/L compound was added to 100  μL of MOLM-13 cell suspension to achieve a final concentration of 20  μmol/L, followed by a 24-h incubation. Compounds with ≥ 90% killing activity were selected as active hits in the preliminary screening, cell survival was measured by CCK8. (B) MOLM-13 cells were treated with 38 compounds (1 μmol/L) that have been first screened for 24 h, cell survival was measured by CCK8. Red triangle represents inhibitors that inhibit p53–MDM2 interactions. (C) Baf3 cells and FLT3-ITD Baf3 cells were treated with six p53–MDM2 interactions inhibitor (1 μmol/L), respectively, for 24 h, cell survival was measured by CCK8. The red box indicates the APG-115 group. (D) Five different AML cells were treated with different concentrations of APG-115 for 24 h. Cell growth was evaluated by CCK8. (E) Baf3 cells, transduced with FLT3-ITD or FLI3-ITD-TKD mutant vectors, were treated with APG-115 (1 μmol/L) for 24 h and cell survival was measured by CCK8. (F) Two healthy control PBMC and primary AML blasts from 5 FLT3-ITD or FLT3-TKD AML patients were exposed to APG-115 (1 μmol/L) for 24 h. Viability was determined by CCK8. ITD-CR (FLT3-ITD complete remission), ITD-RE (FLT3-ITD relapse), and TKD-CR (FLT3-TKD complete remission), ITD (FLT3-ITD initial-onset) denote patient subgroups based on FLT3 mutation status and clinical response. (G) MOLM-13 and MV4-11 cells were exposed to escalating doses of APG-115 for 24 h and then analyzed by immunoblot for FLT3 and p53.

In our search for antitumor compounds targeting MOLM-13 sensitivity, we screened a commercial library and identified 38 compounds that inhibited > 90% of MOLM-13 proliferation at 20 μmol/L (Fig. 1A). To prioritize candidates, we retested compounds at 1 μmol/L, yielding six potent inhibitors that disrupted MDM2–p53 interaction while reducing cell growth by > 60% (Fig. 1B). We next evaluated these six compounds for selective activity against FLT3-ITD cells using Baf3 cells engineered to overexpress FLT3-ITD via lentiviral transduction. APG-115 demonstrated significantly enhanced efficacy against FLT3-ITD Baf3 cells compared to parental cells (Fig. 1C). Expanded analysis across five AML cell lines revealed pronounced sensitivity of MOLM-13 and MV4-11 (wild-type p53/FLT3-ITD) to APG-115, contrasting with minimal effects in FLT3-WT/p53-mutant (THP-1), FLT3-low/p53-null (U937), and FLT3-WT/p53-null (Jurkat) cells (Fig. 1D). Previous research has implicated secondary mutations within the FLT3 tyrosine kinase domain as a prevalent mechanism underlying resistance to tyrosine kinase inhibitors (TKIs), exemplified by F691L, D835Y, and Y842C. APG-115 maintained robust activity against both FLT3-ITD and FLT3-ITD-TKD-mutant cells relative to controls (Fig. 1E and 1F), suggesting broad-spectrum efficacy against FLT3-driven malignancies irrespective of common resistance mutations. Furthermore, APG-115 induced significant degradation of FLT3-ITD protein, offering a novel mechanism to overcome TKI resistance (Fig. 1G). Figure S1A illustrates the structure of APG-115. Through cellular thermal shift assay (CESTA) experiments, we confirmed that the direct target of APG-115 is MDM2 (Fig. S1B and S1C), and its effect is not mediated by broad-spectrum cytotoxicity (Fig. S1D and S1E).

### APG-115 suppresses AML LSCs via cell cycle arrest and differentiation

**Figure 2.**
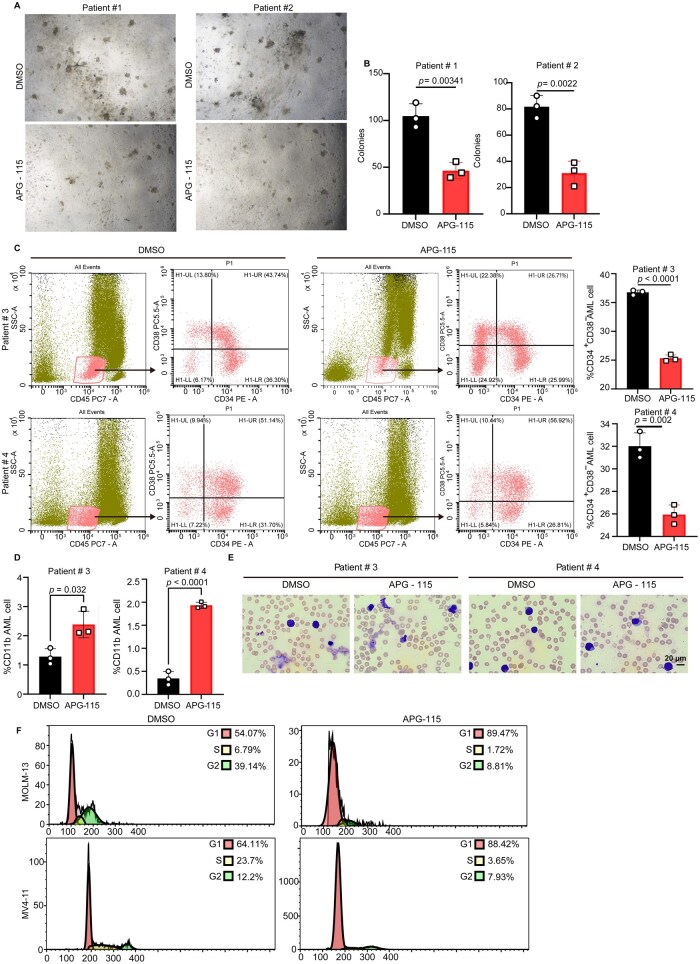
APG-115 suppresses AML LSCs via cell cycle arrest and differentiation. (A, B) Statistical results of colony formation counts in primary AML cells from patients (AML1–2) (*N *= 3; error bars represent mean ± SD). (C) Flow cytometric analysis of the proportion of CD45dim CD34^+^CD38^−^ cells. (D) Flow cytometric detection of CD11b expression in primary bone marrow cells from patients. (E) Wright–Giemsa staining for cell morphology. (F) Flow cytometric analysis of cell cycle distribution in MOLM-13 and MV4-11 cells treated with APG-115 (0.1 μmol/L for 24 h). Representative flow cytometric plots are shown, with *N *= 3 biological replicates.

To deeply investigate the biological effects of APG-115 on FLT3-ITD leukemia stem cells (LSCs), we systematically validated through a series of experiments. APG-115 treatment induced a marked suppression of clonogenic potential in primary AML patient-derived cells, about 60% inhibition rate compared to controls, as quantified by soft agar colony formation assays (Fig. 2A and 2B). Limiting dilution assays yielded consistent results (Fig. S2). Flow cytometric analysis revealed that APG-115 treatment notably reduced the proportion of the stem cell-characteristic CD34^+^CD38^−^ subpopulation (Fig. 2C) and concurrently upregulated the expression of the myeloid differentiation marker CD11b (Fig. 2D). Morphological observation further confirmed that APG-115-treated cells displayed typical differentiated phenotypic features (Fig. 2E). Additionally, cell cycle analysis demonstrated that APG-115 significantly induced cell cycle arrest at the G0/G1 phase, with statistically significant differences (Fig. 2F). APG-115’s dual mechanisms of LSC suppression through cell cycle arrest, differentiation induction, and surface antigen modulation, thereby providing a robust preclinical rationale for its therapeutic development in FLT3-ITD AML.

### TRIM22 mediates p53-mediated inhibition of FLT3-ITD-driven leukemia proliferation

**Figure 3.**
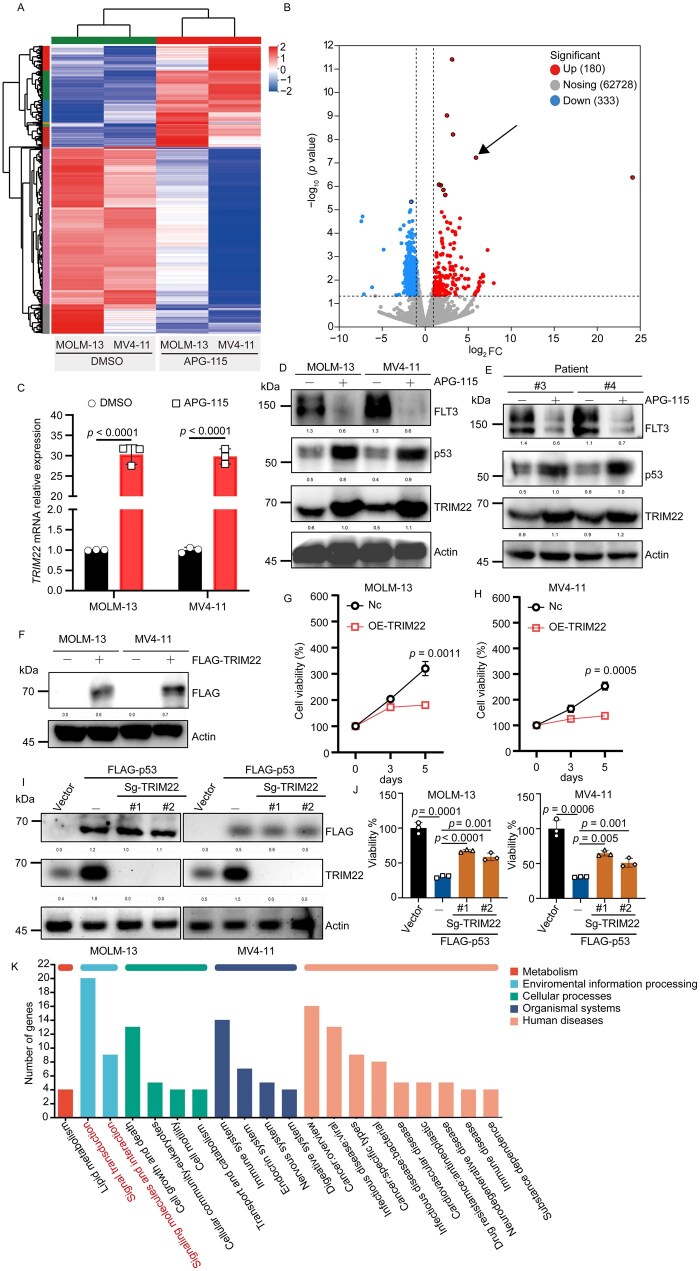
TRIM22 is a functional downstream target of p53. (A, B) Heatmap and volcano plots depicting the differential expression of p53 target genes in MOLM-13 and MV4-11 cells following treatment with APG-115 (0.1 μmol/L, 24 h). (C) qRT-PCR analysis of *TRIM22* expression in MOLM-13 and MV4-11 cells treated with APG-115 (0.1 μmol/L, 24 h). (D) Immunoblotting analysis of indicated proteins in MOLM-13 and MV4-11 cells treated with APG-115 (0.1 μmol/L, 24 h). (E) Immunoblotting analysis of TRIM22 in primary blasts derived from two FLT3-ITD AML patients treated with APG-115 (0.1 μmol/L, 24 h). (F–H) MOLM-13 and MV4-11 cells were transduced with either empty vector (vector) or FLAG-TRIM22-expressing (TRIM22) lentiviral vectors. Cell viability was assessed using the CCK-8 assay. (I) MOLM-13 and MV4-11 cells were transduced with lentivirus expressing either control vector (Ctrl), p53-overexpressing construct (p53 OE), or co-transduced with p53 OE and *TRIM22*-targeting sgRNA to knock out endogenous TRIM22. Stable cell pools were selected and expanded for subsequent assays. (J) Quantification of cell viability measured by CCK-8 assay. (K) KEGG pathway enrichment analysis of significantly downregulated or upregulated genes in APG-115-treated MOLM-13 and MV4-11 cells compared to DMSO controls.

To investigate the regulatory mechanism by which p53 upregulation inhibits FLT3-ITD-driven leukemic cell proliferation, we performed transcriptome profiling (RNA-seq) of FLT3-ITD AML cells treated with the APG-115. Differential expression analysis identified TRIM22 as a significantly upregulated gene (Fig. 3A and 3B). Validation experiments confirmed a consistent increase in both *TRIM22* mRNA (qRT-PCR) and protein levels (Western blot) in two independent FLT3-ITD AML cell lines (Fig. 3C and 3D). Similar results were observed in two primary patient-derived cell (Fig. 3E). More interestingly, we found that APG-115 not only promotes the upregulation of p53 and TRIM22 but also simultaneously induces the degradation of FLT3 protein (Fig. 3D and 3E). Furthermore, TRIM22 overexpression reduces cellular viability (Fig. 3F–H). Genetic ablation of TRIM22 partially rescues the reduced cell viability induced by p53 overexpression (Fig. 3I and 3J). Overexpression of p53 alone is sufficient to induce TRIM22 expression (Fig. S3A). This induction would subsequently lead to the degradation of the FLT3-ITD. Knocking out p53 in the presence of APG-115 would attenuate the induction of TRIM22 expression. Consequently, the apoptosis induced by APG-115 would be alleviated (Fig. S3B). Depletion of TRIM22 would compromise the effect of APG-115 in AML cells (Fig. S3C). This result indicates that TRIM22 is a crucial downstream effector mediating the therapeutic action of APG-115, possibly through facilitating the degradation of target proteins like FLT3-ITD. Consistent with these findings, signaling pathway profiling of APG-115-treated cells further revealed significant functional perturbations in membrane-associated signal transduction pathways (Fig. 3K). Those findings implicate TRIM22 as a potential mediator in p53-mediated suppression of FLT3-ITD AML cell proliferation, warranting further investigation into its role in the p53-FLT3 signaling axis.

### TRIM22 facilitates proteasomal degradation of FLT3-ITD via ubiquitination

**Figure 4.**
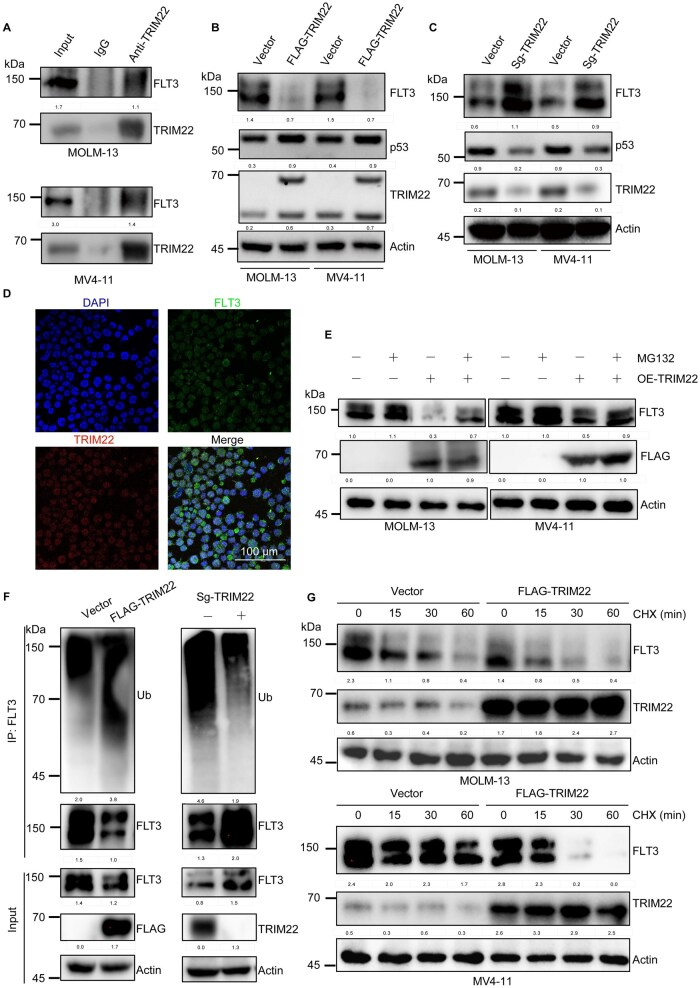
TRIM22 interacts with and regulates FLT3-ITD stability. (A) Protein lysates of MOLM-13 and MV4-11 cells were immunoprecipitated with TRIM22 antibody and then immunoblotted for TRIM22 and FLT3. (B, C) MOLM-13 and MV4-11 cells transduced with empty (vector) or FLAG-TRIM22-expressing lentiviral vectors, or a lentiviral vector expressing sgTRIM22. Immunoblot of indicated proteins. (D) Representative immunofluorescence images of FLT3-ITD (green) and TRIM22 (red) in MOLM-13. (E) MOLM-13 and MV4-11 cells were transfected with FLAG-TRIM22. After 48 h, the cells were treated with MG132 for 6 h, followed by cell lysis and then immunoblotted for indicated proteins. (F) MOLM-13 cells transduced with FLAG-TRIM22-expressing lentiviral vectors or sgRNA. Protein lysates were immunoprecipitated with FLT3 antibody and then immunoblotted for indicated proteins. (G) MOLM-13 and MV4-11 cells transduced with vector or TRIM22-expressing lentiviral vectors. Immunoblot of FLT3 and TRIM22 after CHX (100 μg/mL) treatment for indicated minutes.

We systematically elucidated the molecular mechanism by which TRIM22, a downstream effector of p53, regulates FLT3-ITD protein stability. Through endogenous co-immunoprecipitation assays, we demonstrated that TRIM22 directly interacts with FLT3-ITD (Fig. 4A). Functional analyses revealed that TRIM22 knockdown significantly increased FLT3-ITD protein abundance, whereas its overexpression reduced protein levels, indicating post-translational regulation (Fig. 4B and 4C). The immunofluorescence staining also showed that FLT3-ITD co-localized with TRIM22 in the cytoplasm in MOLM-13 cells (Fig. 4D). MG132 can reverse the degradation of FLT3-ITD induced by TRIM22 overexpression (Fig. 4E). To define the underlying mechanism, we assessed FLT3-ITD ubiquitination status. TRIM22 depletion decreased total ubiquitination levels, while TRIM22 overexpression increased total ubiquitination (Fig. 4F). Strikingly, protein stability assays showed that TRIM22 overexpression shortened FLT3-ITD half-life (Fig. 4G), consistent with reduced ubiquitination. Mechanistically, our data suggest that p53 activation transcriptionally upregulates TRIM22 expression, which subsequently acts as an E3 ubiquitin ligase to promote FLT3-ITD ubiquitination and degradation.

### FLT3 inhibition leads to the downregulation of p53 via FLT3-ITD signaling inhibition in AML cells

**Figure 5.**
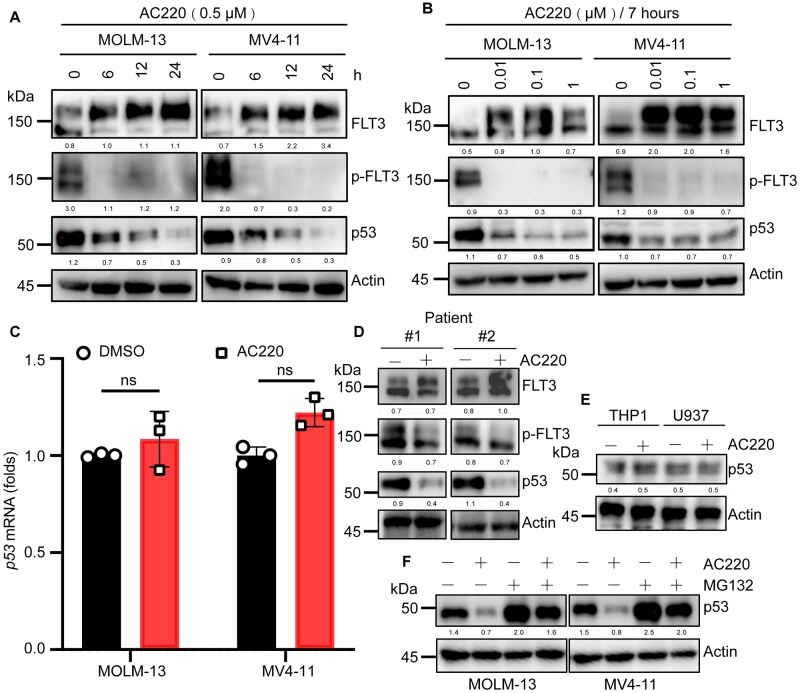
AC220 downregulates p53 protein in FLT3-ITD AML cells. (A, B) Immunoblot analysis of indicated protein expression in MOLM-13 and MV4-11 cells treated with varying concentrations and durations of AC220. (C) Quantitative real-time PCR (qRT-PCR) quantification of *TP53* mRNA levels in MV4-11 and MOLM-13 cells following treatment with 10 nmol/L AC220 for 24 h (*n *= 3 biological replicates; data are mean ± SD). (D) Immunoblot detection of p53 protein in primary blasts isolated from two FLT3-ITD-positive AML patients after 24-h treatment with 100 nmol/L AC220. (E) Immunoblot analysis of p53 expression in THP1 and U937 cells treated with 100 nmol/L AC220 for 24 h. (F) Immunoblot analysis of indicated proteins in MV4-11 and MOLM-13 cells treated with AC220 or MG132, to assess potential regulatory mechanisms.

Our investigations revealed that treatment with AC220 exerted a time-dependent and concentration-dependent inhibitory effect on the expression of p53 protein in MV4-11 and MOLM-13 cells harboring FLT3-ITD mutations (Fig. 5A and 5B). However, this inhibitory effect was not observed at the mRNA expression level of p53 (Fig. 5C). In a similar vein, treatment with AC220 in primary human FLT3-ITD AML samples resulted in a significant downregulation of p53 protein (Fig. 5D). Importantly, AC220 had no discernible impact on the levels of p53 protein in cell lines such as THP1, and U937, which do not express FLT3-ITD (Fig. 5E). Collectively, these findings provide compelling evidence that the downregulation of p53 is a direct consequence of FLT3-ITD signaling inhibition, rather than an off-target effect (Fig. 5E). In addition, the reduction in p53 protein levels was found to be reversible upon treatment with proteasome inhibitors, suggesting that p53 degradation likely occurs via the ubiquitination pathway (Fig. 5F). This mechanism of p53 regulation in response to FLT3-ITD inhibition underscores the intricate interplay between FLT3 signaling and the tumor suppressor p53 in AML cells.

### APG-115 enhances AC220-mediated elimination of FLT3-ITD AML *in vitro* and *in vivo*

**Figure 6.**
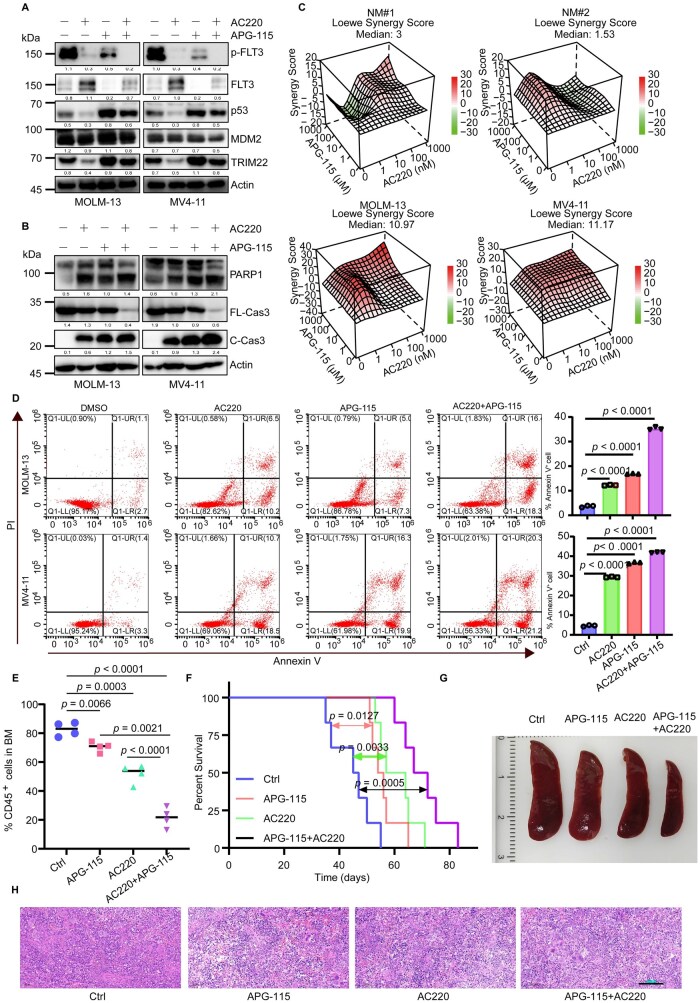
APG-115 enhances AC220-mediated elimination of FLT3-ITD AML. (A, B) Immunoblot of indicated proteins in MOLM-13 and MV4-11 cells after treatment as indicated (10 nM AC220; 0.1 μM APG-115) for 24 h. (C) Peripheral blood mononuclear cells (PBMCs) from two healthy donors and MOLM-13, MV4-11 were treated with gradient concentrations of AC220 and APG-115, either as monotherapy or in combination for 24 h. Cell viability was measured by CCK8, and the Synergy Index was calculated online (synergyfinder.org). (D) MOLM-13 and MV4-11cells were treated 10 nmol/L AC220 or 0.1 μmol/L APG-115 for 24 h. Cell apoptosis was analysed by Annexin V/PI staining. (E) Percentage of CD45^+^ MV4-11 cells in the bone marrow of mice (*n *= 4 per group; mean ± SD). (F) Kaplan–Meier survival of mice treated with different chemicals is displayed, respectively (*n *= 6 per group). (G, H) Representative macroscopic and H&E staining images of spleens.

Mechanistic investigations demonstrated that APG-115, through significantly upregulating the p53/TRIM22 co-regulated gene network (Fig. 6A), effectively reversed the AC220-mediated inhibition of the p53 signaling pathway. *In vitro* experimental data further revealed that this combination therapeutic regimen induced a marked increase in apoptosis rate and a reduction in cell viability in the MOLM-13 and MV4-11cells (Fig. 6B–D). Notably, this selective cytotoxicity persisted in primary FLT3-ITD AML patient-derived cells while exhibiting no significant toxicity toward human peripheral blood mononuclear cells (Fig. 6C). Preclinical validation in FLT3-ITD AML cell-derived xenograft (CDX) models demonstrated that the combination therapy group displayed a significantly reduced bone marrow tumor burden (Fig. 6E) and an extension in median survival (Fig. 6F). APG-115 showed no significant toxicity in mice (Fig. S4). Histopathological analysis showed that the combination therapy group exhibited markedly reduced leukemia cell infiltration in the splenic tissue, along with a characteristic cell enlargement phenotype; assessment of splenic tissue architecture indicated that the combination therapy group more closely resembled normal physiological status compared with the single-agent and control groups (Fig. 6G and 6H).

## Discussion

**Figure 7.**
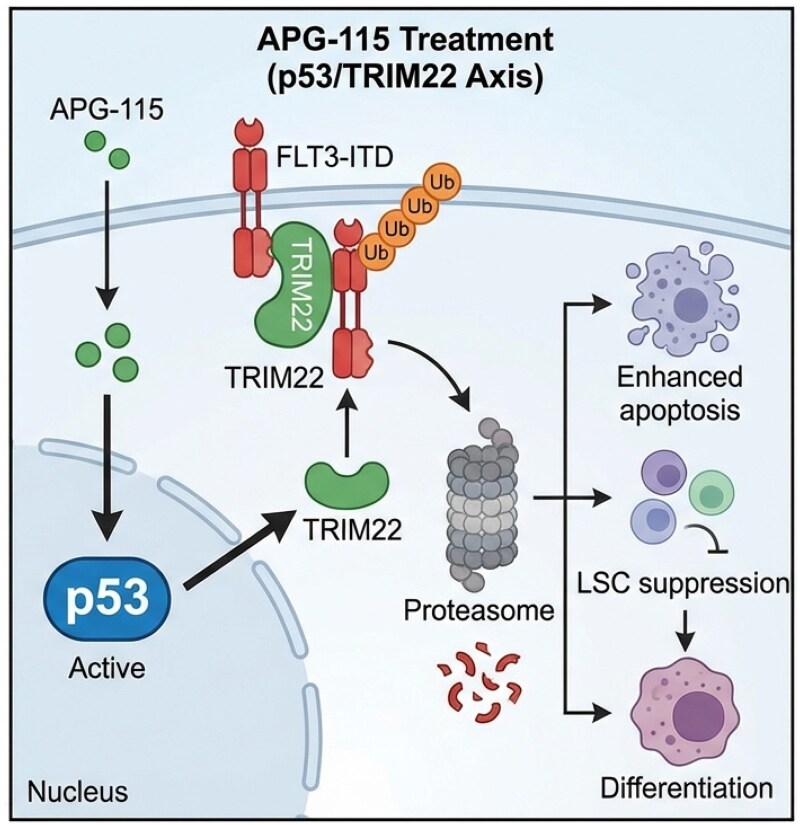
Graphical summary of the degradation of FLT3-ITD mediated by TRIM22 and the contribution to resistance of FLT3 inhibitors. FLT3-ITD signaling induces oncogenic networks and resistance to FLT3 tyrosine kinase inhibitors (TKIs). APG-115 is a small-molecule compound that causes the p53 signaling pathway to be activated and thus increase the expression of TRIM22. TRIM22 facilitates ubiquitination and degradation of FLT3-ITD, which inhibits its oncogenic signaling activity. The result of this process is an increase in apoptosis of leukemia stem cells, cell-cycle arrest and myeloid differentiation, and reduction in clonogenic potential. All these effects lead to better survival and less tumor burden.

This study identified APG-115 as a potent and selective inhibitor of FLT3-ITD-positive AML cells through systematic compound screening, and elucidated its molecular mechanism via the p53/TRIM22 signaling axis to induce ubiquitination-mediated degradation of FLT3-ITD protein. Compared with existing FLT3 tyrosine kinase inhibitors, APG-115 exhibits a unique dual mechanism of action: it both activates the classical p53 pathway to induce cell cycle arrest and apoptosis and engages TRIM22 to activate the ubiquitin–proteasome system, specifically promoting FLT3-ITD protein degradation. Notably, APG-115 not only effectively eliminates leukemia stem cells (LSCs) but also overcomes common TKI resistance mutations within the kinase domain (such as F691L, D835Y, and Y842C), offering a novel precision therapeutic strategy for AML (Fig. 7).

TRIM22 is a direct transcriptional target of p53 and mediates p53’s tumor-suppressive functions [23–25]. In leukemia, TRIM22 overexpression inhibits the clonogenic growth of monoblastic U937 cells, and its knockdown enhances cell proliferation, suggesting a potential tumor-suppressive role in myeloid malignancies [20]. However, its specific function in FLT3-ITD AML remains unexplored. TRIM22 functions as an E3 ubiquitin ligase via its RING domain, catalyzing ubiquitin conjugation to substrate proteins [26]. Its substrate specificity exhibits tissue-context dependency. In glioma, TRIM22 stabilizes the pro-survival protein Bcl-2 through non-degradative ubiquitination [27]. In gastric cancer, it suppresses proliferation by binding to Smad [28]. This context-dependent regulatory capacity provides a theoretical rationale for targeting TRIM22 to degrade oncoproteins like FLT3-ITD. A pivotal discovery in this study is the role of TRIM22 in mediating FLT3-ITD ubiquitination and degradation. TRIM22 overexpression mimicked the effects of APG-115, reducing FLT3-ITD half-life and impairing cell viability. This positions TRIM22 as a critical effector linking p53 activation to FLT3-ITD turnover, offering a new therapeutic node for intervention, which aligns with the findings of Seipel et al. [29] regarding the synergistic anti-leukemic effects of MDM2 inhibitors and FLT3 inhibitors. The specificity of TRIM22 for FLT3-ITD (vs. FLT3-WT) remains to be fully elucidated but may involve unique structural features of the ITD mutant, which could alter protein–protein interaction interfaces. Recent advances in FLT3-ITD detection methodologies, such as modified PCR assays [30], could facilitate further investigations into how ITD mutations influence protein stability and interactome dynamics.

Although MDM2 inhibitors demonstrate anti-leukemic activity in AML [31, 32], we establish that APG-115 exhibits potent and selective growth suppression specifically in FLT3-ITD AML cell lines (MOLM-13, MV4-11) while showing minimal effects on FLT3-WT cells. Crucially, APG-115 retains efficacy against FLT3-ITD variants harboring tyrosine kinase domain (TKD) resistance mutations (e.g. F691L, D835Y), which typically confer resistance to FLT3 tyrosine kinase inhibitors such as sorafenib and gilteritinib [7, 33, 34]. Compared to conventional tyrosine kinase inhibitors that primarily inhibit FLT3 kinase activity and frequently develop acquired resistance due to secondary mutations in the tyrosine kinase domain, APG-115 exhibits a critical mechanistic advantage. APG-115-mediated p53 stabilization, induces proteasomal degradation of FLT3-ITD through TRIM22, rather than merely suppressing kinase activity. This degradation-dependent strategy may circumvent acquired resistance observed with FLT3 inhibitor monotherapy [13, 35]. This finding carries significant clinical implications since clinically deployed FLT3 inhibitors (e.g. quizartinib, gilteritinib) show limited efficacy against TKD-mutant clones, a recognized clinical challenge. These results align with emerging studies emphasizing mechanistically distinct approaches beyond kinase inhibition, such as targeted protein destabilization or pathway modulation, to combat FLT3-ITD-driven malignancies, as demonstrated in recent protein degradation strategies against viral pathogens and oncoproteins [36, 37].

Our data demonstrate that APG-115 exerts multifunctional inhibitory effects on leukemia stem cells (LSCs), significantly impairing their colony-forming capacity by about 60%, inducing G0/G1 cell cycle arrest, and promoting differentiation as evidenced by upregulation of CD11b. Critically, APG-115 significantly reduces the primitive CD34^+^CD38^−^ LSC compartment, indicating potent targeting of LSC self-renewal—a key factor in AML relapse. This differentiation-promoting effect aligns with the reported actions of other MDM2 inhibitors [38]. However, our findings reveal a distinct molecular mechanism underpinning APG-115’s efficacy against LSCs. Notably, we propose that APG-115 activates the p53-TRIM22 axis, which consequently promotes the degradation of the FLT3-ITD oncoprotein. This mechanism is strongly supported by the established critical role of FLT3 in LSC maintenance: FLT3 knockout has been shown to completely eradicate LSCs in FLT3-ITD AML while sparing normal hematopoietic stem cells (HSCs), validating FLT3 as a highly specific therapeutic target for these LSCs [39]. Therefore, the profound reduction in functional LSCs observed following APG-115 treatment (both phenotypically and functionally) can be mechanistically linked to the p53–TRIM22 axis-mediated degradation of FLT3-ITD. This delineates a novel strategy for targeting the LSC reservoir responsible for relapse in FLT3-ITD AML.

Combined targeting of FLT3 and p53 pathways exhibited significant synergistic anti-leukemic effects. The co-administration of APG-115 and AC220 markedly activated the p53/TRIM22 axis, promoted FLT3-ITD degradation, and enhanced apoptosis. This finding aligns with the dual inhibition strategy proposed by Jiang et al. [40] (e.g. CHK1/FLT3 co-inhibition), which overcomes resistance by simultaneously targeting multiple key pathways. However, our study further clarified the central role of TRIM22 in this synergistic effect. Notably, Short et al. [41] found that the BCL-2 inhibitor venetoclax, in combination with chemotherapeutic agents, is effective against FLT3-ITD AML, but whether TRIM22 regulates the expression of BCL-2 family proteins remains to be validated. Combined targeting of FLT3 and p53 pathways synergistically activates TRIM22-mediated FLT3-ITD degradation, overcoming the limitations of monotherapy. This strategy is not only applicable to FLT3-ITD AML but may also extend to other hematologic malignancies dependent on tyrosine kinase mutations. Future clinical translation studies should prioritize the tolerability of combination therapies and the mechanisms to overcome resistance.

In summary, this study unveiled the feedback regulatory mechanism of the p53–TRIM22–FLT3-ITD axis, providing a novel therapeutic target for FLT3-ITD AML. This bidirectional axis creates a vulnerability where APG-115 simultaneously blocks FLT3-ITD-induced p53 degradation and utilizes restored p53 activity to destroy FLT3-ITD via TRIM22, culminating in synergistic lethality with FLT3 inhibitors and providing a novel mechanism to overcome TKI resistance in AML.

## Research limitations

The limitations of this study include the incomplete elucidation of the specific E3 ligase mechanisms by which FLT3-ITD regulates p53 ubiquitination. Additionally, while the efficacy of combination therapy was validated in cell lines and CDX models, the lack of PDX models may limit the predictive value for clinical translation. Abe et al. [42] demonstrated that FLT3-ITD regulates cell proliferation and drug resistance through the p21/Pbx1 axis, and whether TRIM22 intersects with this pathway requires further investigation. Future research could focus on developing TRIM22 small-molecule agonists or optimizing delivery systems for p53 stabilizers (e.g. the LNP-based circRNA delivery strategy employed by Li et al. [43]) to enhance therapeutic efficacy.

## Methods

### Research ethics

The study was approved by the Ethics Committee of Medical Ethics Committee of the First Affiliated Hospital of Xi’an Jiaotong University (XJTU1AF2023LSK-080). All human tissues acquisition complied with institutional guidelines and were conducted following documented written informed consents. Animal studies were performed in accordance with protocols approved by the Health Science Center of XJTU Approval for Research Involving Animals. Experiments were performed under the approval of the Experimental Animal Ethical Committee at Xi’an Jiao Tong University School. (Approval No: 2022QN-11).

### Cell culture

Human leukemia cell lines (MV4-11, MOLM-13, U937, THP-1, Jurkat) and mouse lymphocyte-derived BaF3 variants were cultured in complete media (IMDM or RPMI-1640) supplemented with FBS, antibiotics, and IL-3 (for BaF3 cells). All cells were maintained at 37°C with 5% CO_2_.

### Antibodies and reagents

Anti-FLT3 (clone BV10A4H2), mouse monoclonal antibody (mAb), Alexa Fluor^®^ 488-conjugated (Cell Signaling Technology, USA, Cat No. 23991S); Anti-Phospho-FLT3 (Tyr591) (clone 8F2), rabbit mAb (Cell Signaling Technology, USA; Cat No. 3462); Anti-CD45, PC7-conjugated (Beckman Coulter, USA; Cat No. IM3548); Anti-CD34, PE-conjugated (Beckman Coulter, USA; Cat No. A07776); Anti-CD38, PC5.5-conjugated (Beckman Coulter, USA; Cat No. B49199); Anti-TRIM22 rabbit polyclonal antibody (Proteintech, China; Cat No. 13744-1-AP); Anti-p53 mouse mAb (Proteintech, China; Cat No. 60283-2-Ig). MethoCult™ H4034: Methylcellulose-based semisolid medium with recombinant human cytokines (STEMCELL Technologies, Canada; Cat No. H4436); Ficoll-Paque™ PLUS density gradient medium (cytometric-grade; GE Healthcare, USA; Cat No. 17–1440-02).

### Compound library screening

Primary screening: MOLM-13 cells were seeded in 96-well plates at 5 × 10^3^ cells/well and treated with a commercial compound library (MCE Library-Detailed Information-HY-LD-000003419-2-Jun 16, 2022) 20 μmol/L final concentration for 72 h. DMSO (0.1%) served as vehicle control. Cell proliferation was assessed using CCK-8 assay. Compounds showing > 90% inhibition were selected for secondary screening. Secondary screening: Selected compounds were retested at 1 μmol/L in MOLM-13 cells. Proliferation inhibition was measured as above, and MDM2–p53 interaction disruption was evaluated using CCK-8. Compounds demonstrating > 60% growth suppression with concurrent target inhibition were advanced. Selectivity validation: BaF3 cells engineered to overexpress FLT3-ITD via lentiviral transduction were subjected to treatment with candidate compounds. IC_50_ values were calculated for both FLT3-ITD-BaF3 and parental BaF3 cells using nonlinear regression (GraphPad Prism v9.0).

### Colony-forming unit assay

Primary AML blasts were cultured in MethoCult^®^ H4436 for 10 days. Colony-forming unit (CFUs) (≥ 50 cells) were counted under microscopy. Data analyzed using GraphPad Prism 9.

### Cell cycle analysis by flow cytometry

Cells were fixed in ethanol, stained with PI/RNase A, and analyzed by flow cytometry (488 nm excitation).

### Mononuclear cell isolation (Ficoll-Paque™ density gradient centrifugation)

PB/BM samples from FLT3-ITD AML patients were processed via Ficoll-Paque™ gradient centrifugation. MNCs were isolated, lysed (ACK buffer), and resuspended in IMDM.

### Plasmid construction

The open reading frame of human TRIM22 was subcloned into the pLVX-Puro retroviral vector (Clontech, USA) to generate pLVX-Puro-FLAG-TRIM22. *TRIM22* sgRNA sequences (forward: 5′-TGGCCAGATGCCGATTAGGT-3′; reverse: 5′-AAACACCTAATCGGCATCTGGCCAC-3′) were designed using the CRISPick algorithm.

### Immunofluorescence staining

Cells were fixed with 4% paraformaldehyde (PFA) for 15 min, permeabilized with 0.5% Triton X-100 in PBS for 10 min, and blocked in 2% BSA/PBS for 1 h at RT. Primary antibodies were incubated overnight at 4°C, followed by Alexa Fluor 594-conjugated secondary antibodies (1:500; Invitrogen, USA) for 1 h at RT. Nuclei were counterstained with DAPI (0.1 μg/mL). Images were acquired using a Leica confocal microscope (Leica Microsystems, Germany) with LAS AF Lite software.

### CETSA

Cells were treated with APG-115 or control, the cell suspensions were then aliquoted and subjected to brief heat treatment at a defined temperature gradient or with varying drug concentrations, followed by rapid cooling and lysis. Finally, the stability of the target protein under different temperatures was detected by Western blot.

### CDX model

MV4-11 (2 × 10^6^ cells) were injected into 6-week-old female NOD-SCID mice via tail vein. After confirmation of human leukemia engraftment in murine peripheral blood (> 5% human CD45^+^ cells), treatment was initiated as indicated (quizartinib, 10 mg/kg daily for 4 weeks, gavage; APG-115, 25 mg/kg daily, gavage). We defined the first occurrence of endpoint (moribundity or death) in the control group as the sampling time point. At this time, four mice were randomly selected from each group (including the control and treatment groups), and the proportion of human CD45^+^ MV4-11 cells in the bone marrow was assessed by flow cytometry to evaluate tumor cell infiltration. Subpopulations and immunophenotype of human CD45^+^ cells were evaluated by labeling with anti-human CD33. The remaining mice continued to receive treatment until they reached the endpoint (moribundity or death), and were used exclusively for constructing Kaplan–Meier survival curves and performing survival analysis.

### Statistics analysis

Data visualizing and statistical analysis were performed using GraphPad Prism software. Survival comparisons were performed using the Kaplan–Meier/log-rank test. Other differences between experimental groups were analyzed using a paired or unpaired Student’s *t*-test and 2-way analysis of variance with multiple testing. A *P*-value 0.05 was considered significant in all experiments.

## Supplementary Material

lnag017_Supplementary_Data

## Data Availability

The data that support the findings of this study are available from the corresponding author upon reasonable request.
