# Supplementary material for: TRIM22-mediated ubiquitin-dependent degradation of FLT3-ITD overcomes TKI resistance in acute myeloid leukemia
Source: Life Med. 2026 May 14;5(3):lnag017. doi: 10.1093/lifemedi/lnag017 (PMC13319326; doi:10.1093/lifemedi/lnag017)
Supplement: lnag017_Supplementary_Data [file lnag017_supplementary_data.zip › 096_SI.docx]

TRIM22-mediated ubiquitin-dependent degradation of FLT3-ITD overcomes TKI resistance in acute myeloid leukemia

Meng Liu^1,#^, Xiaoqian Wang^1,#^, Xiaqin He^1,#^, Xiaoguang Xu^2^, Jing Chen^2^, Juan Liu^2^, Shan Jiang^1^, Sijia Li^1^, Si Wang^1^, Yuanyuan Liu^1^, Jing Feng^1^, Xin Xu^1^, Miaoyin Luo^1^, Zhiyi Li^2^, Yining Zhang^3^, Yingli Wu^4,*^, Jian Hu^1,*^, Xiaoqin Wang^1,*^

^1^Department of clinical laboratory, The First Affiliated hospital of Xi'an Jiaotong University, Xi'an 710061, China
^2^Department of hematology, The First Affiliated hospital of Xi'an Jiaotong University, Xi'an 710061, China

^3^Department of blood transfusion, The First Affiliated hospital of Xi'an Jiaotong University, Xi'an 710061, China
^4^Hongqiao International Institute of Medicine, Shanghai Tongren Hospital/Faculty of Basic Medicine, Chemical Biology Division of Shanghai Universities E-Institutes, Key Laboratory of Cell Differentiation and Apoptosis of the Chinese Ministry of Education, Shanghai Jiao Tong University School of Medicine, Shanghai 200025, China

^#^These authors contributed equally to this work.

*Correspondence: wxq1493722680@xjtufh.edu.cn (X.W.), [hobbyhujian@sina.com](mailto:hobbyhujian@sina.com) (J.H.), [wuyingli@shsmu.edu.cn](mailto:wuyingli@shsmu.edu.cn) (Y.W.).

**Figure S1. Validation of APG-115’s direct target engagement and apoptosis induction.**

(A) This is the molecular structure of APG-115. CESTA was performed to detect direct drug-target engagement. Cells were treated with APG-115 or vehicle control, subjected to a temperature gradient (B) or dose gradient (C), and then lysed. The stability of the MDM2 protein across temperatures was analyzed by Western blot. (D, E) Cells were treated with a range of APG-115 concentrations, and apoptosis was assessed using Annexin V and propidium iodide (PI) dual staining followed by flow cytometry analysis at the indicated time points.

**Figure S2. APG-115 suppresses AML LSCs.**

(A, B) Mononuclear cells from two patients were isolated, subjected to serial dilution, treated with APG-155, and then analyzed by ELDA to calculate stem cell frequency.

**Figure S3. TRIM22 mediates the therapeutic effect of APG-115 in a p53-dependent manner.**

(A) Cells were transfected to overexpress p53, and TRIM22 and FLT3-ITD protein levels were assessed by Western blot. (B) p53 was knocked out using CRISPR/Cas9 in cells treated with or without APG-115. Apoptosis was assessed by Annexin V and PI staining followed by flow cytometry, and TRIM22 expression was analyzed by western blot. (C) TRIM22 was depleted using CRISPR/Cas9 in cells treated with APG-115, and the effect on cell viability or apoptosis was measured.

**Figure S4. APG-115 demonstrates a favorable safety profile *in vivo* with no significant toxicity observed in mice.**

To assess the *in vivo* safety of APG-115, mice were administered either the compound or a vehicle control every two days for 23 consecutive days. (A) Mouse body weight was monitored throughout the treatment period. (B, C) At the endpoint, blood samples were collected for serum biochemistry analysis. (D) Major organs (heart, liver, spleen, lungs, and kidneys) were harvested, fixed, sectioned, and stained with Hematoxylin and Eosin (H&E) for histopathological examination.
