# Supplementary figures and images for: TRIM22-mediated ubiquitin-dependent degradation of FLT3-ITD overcomes TKI resistance in acute myeloid leukemia
Source: Life Med. 2026 May 14;5(3):lnag017. doi: 10.1093/lifemedi/lnag017 (PMC13319326; doi:10.1093/lifemedi/lnag017)

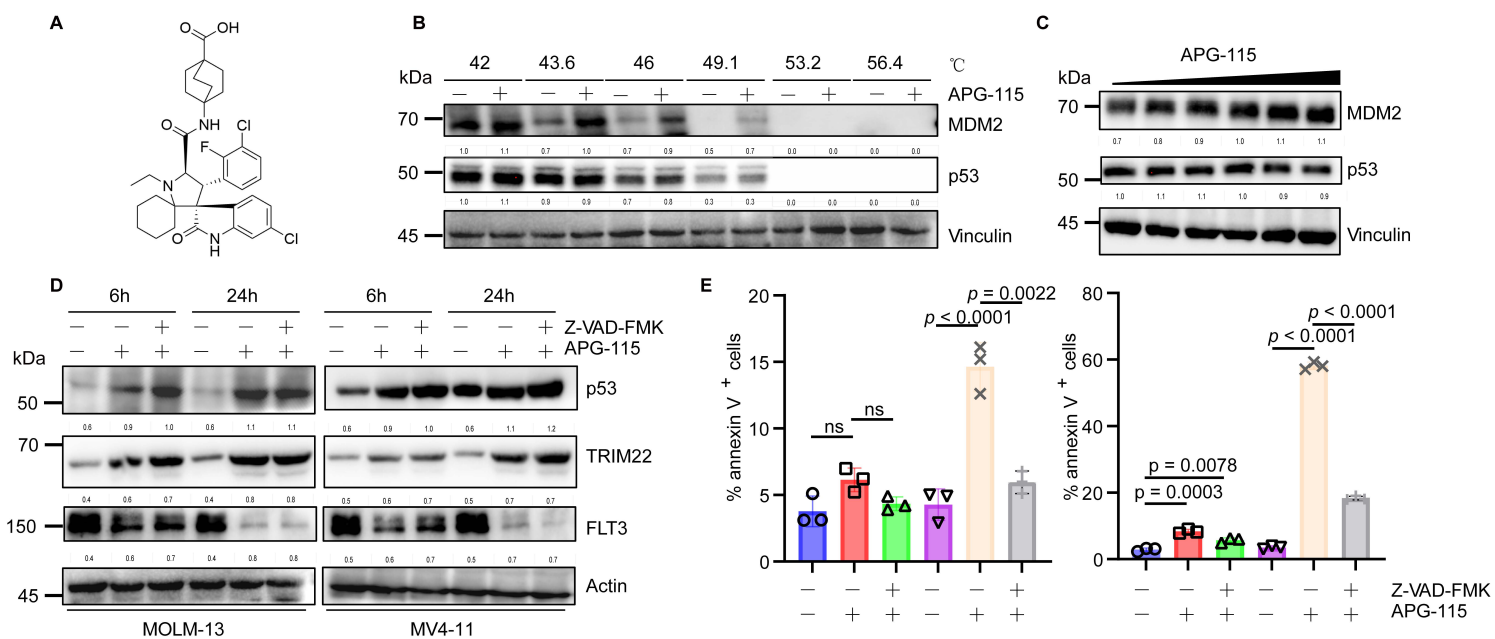

Supplement: lnag017_Supplementary_Data [file lnag017_supplementary_data.zip › s_figure-1_R.pdf]

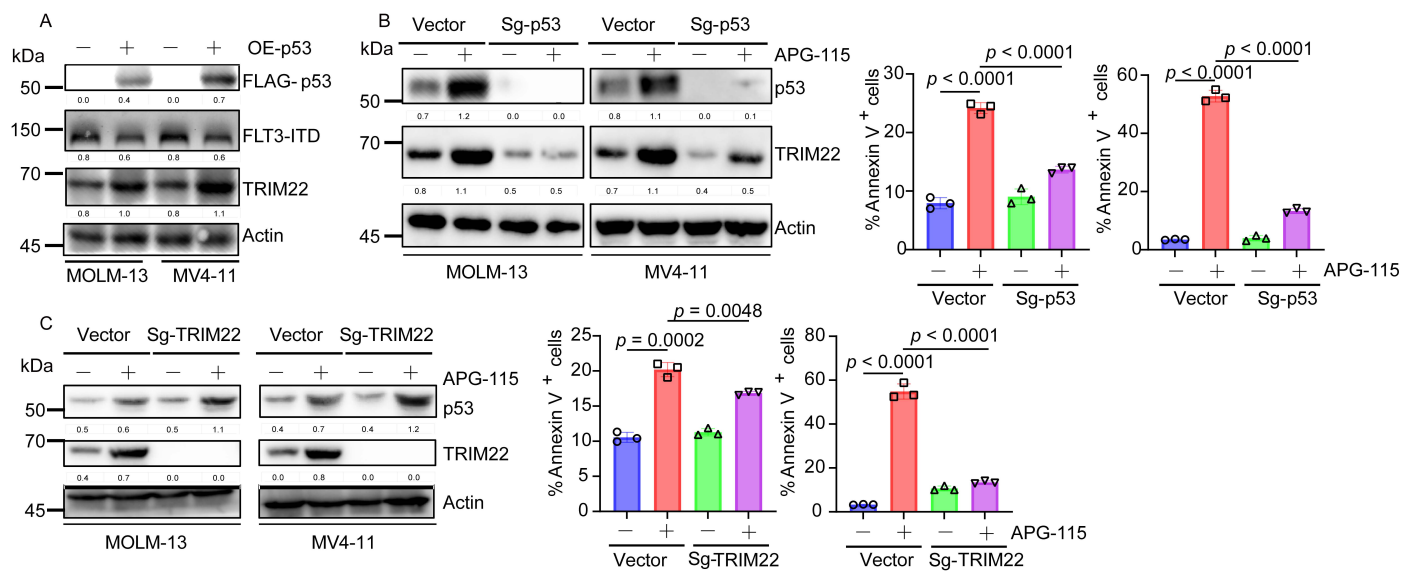

Supplement: lnag017_Supplementary_Data [file lnag017_supplementary_data.zip › s_figure-3_R.pdf]

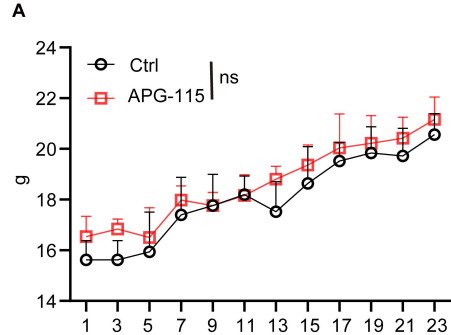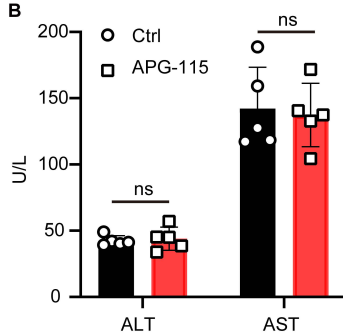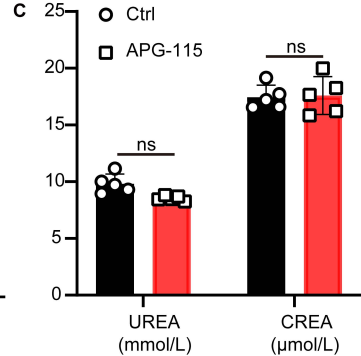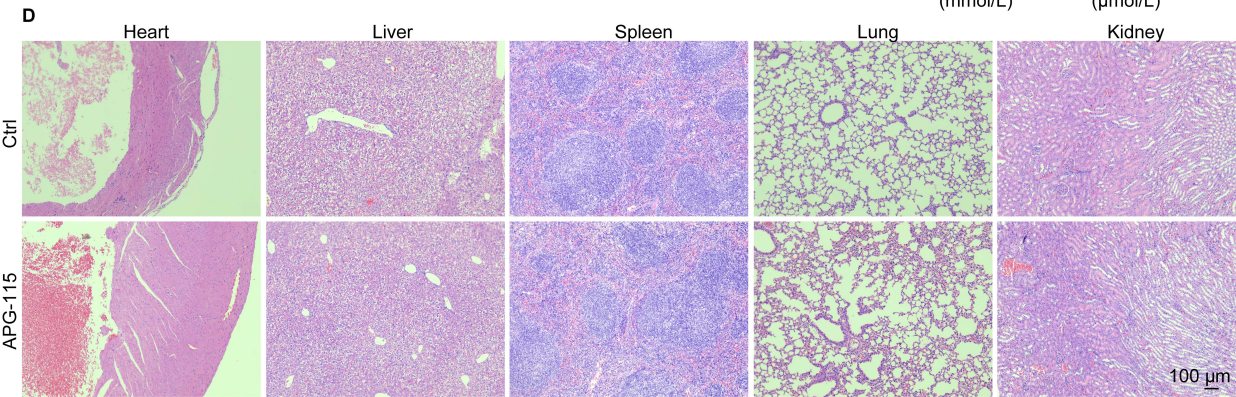

Supplement: lnag017_Supplementary_Data [file lnag017_supplementary_data.zip › s_figure-4_R.pdf]

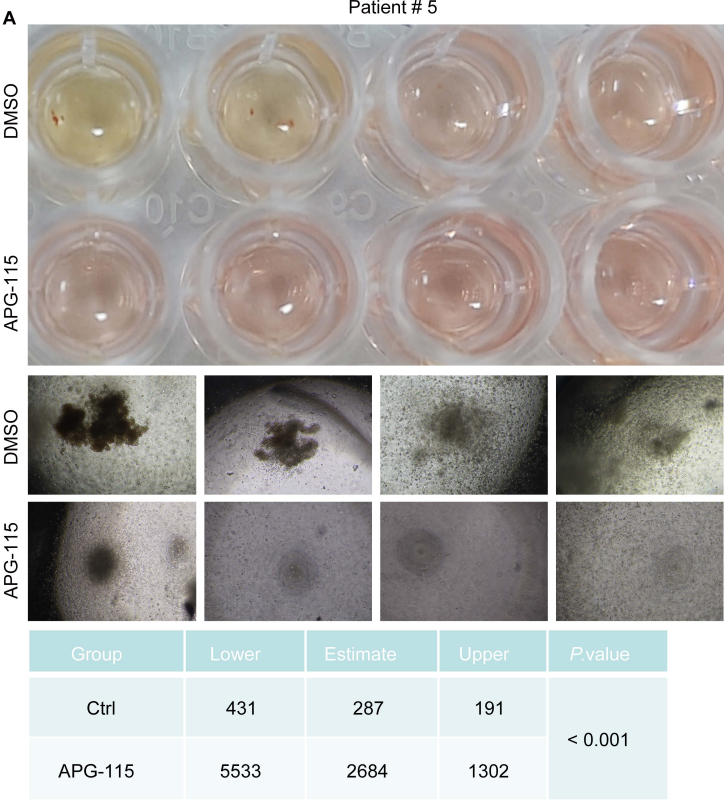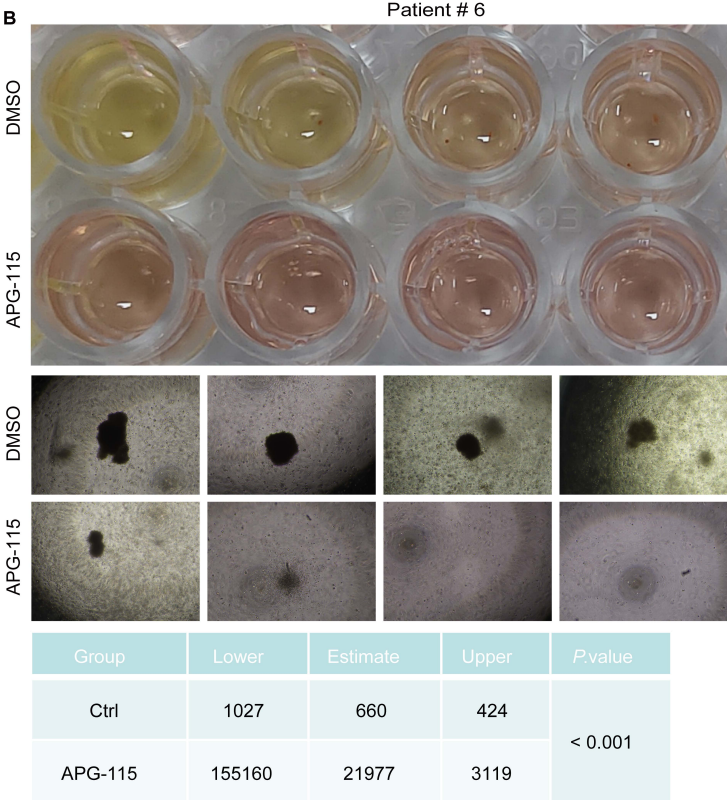

Supplement: lnag017_Supplementary_Data [file lnag017_supplementary_data.zip › s_figure-2_R.pdf]
